# Identifying the *Zheng* in psoriatic patients based on latent class analysis of traditional Chinese medicine symptoms and signs

**DOI:** 10.1186/1749-8546-9-1

**Published:** 2014-01-03

**Authors:** Xuesong Yang, Virasakdi Chongsuvivatwong, Sanguan Lerkiatbundit, Jianzhou Ye, Xiaoyong Ouyang, Enpin Yang, Hutcha Sriplung

**Affiliations:** 1 Dermatology Department, Yunnan Provincial Hospital of Traditional Chinese Medicine, Kunming 650011, China; 2 Epidemiology Unit, Faculty of Medicine, Prince of Songkla University, Hat Yai, Songkhla 90110, Thailand; 3 Faculty of Pharmacy, Prince of Songkla University, Hat Yai, Songkhla 90110, Thailand

## Abstract

**Background:**

There are approximately five *Zhengs* reported in psoriatic patients. Systematic data collection and proper analysis for the classification of psoriasis have been lacking. This study aims to cluster the *Zhengs* in psoriatic patients based on the application of a checklist of traditional Chinese medicine (TCM) symptoms and signs followed by latent class analysis (LCA).

**Methods:**

A cross-sectional study of 507 psoriatic patients aged above 10 years was performed in Yunnan Provincial Hospital of TCM and the First Affiliated Hospital of Kunming Medicine University from October 2010 to September 2011 using a TCM symptoms and signs checklist obtained from 16 TCM experts by the Delphi technique. LCA was applied to obtain the best fitted model for clustering of symptoms and signs that can be interpreted as underlying *Zhengs* of psoriasis.

**Results:**

The LCA identified three *Zhengs*: *dampness-heat Zheng* (35.1%); *blood heat Zheng* (34.7%); and *yin deficiency* and *blood dryness Zheng* (30.2%). The first *Zheng* was associated with winter, the second with male sex, old age, smoking, and drinking alcohol, and the third with outpatient status, which reflected a mild disease course.

**Conclusions:**

In this study, 507 psoriasis patients were clustered into three *Zheng*s, which had different associated factors.

## Introduction

Psoriasis is a chronic life-long inflammatory disease that primarily affects the skin [1], and has a prevalence in the general population that ranges between 0.6% and 4.8% [2]. The disease is associated with an increased risk of metabolic syndrome, diabetes, hypertension, and cardiovascular mortality [3,4], suicidality [5], depression and anxiety [6], and low employment, income, and quality of life [7]. In the United States, psoriasis was estimated to have incurred 6.7 billion United States dollars (USD) of direct costs and 4.5 billion USD of indirect costs, including administration, personnel, and security costs, in 2010 [8]. The total annual costs for psoriasis treatment amounted to approximately 350–510 million USD in Switzerland in 2004 [9].

The diagnosis of psoriasis in traditional Chinese medicine (TCM) was first recorded by Cao Yuan Fang in the Sui Dynasty (AD 610) as “dry ringworm” (*ganxuan*), with descriptions of the symptoms, signs, and pathogenic mechanism of psoriasis, in his book “Zhu Bing Yuan Houlun” [10]. In the Qing dynasty (AD 1665), psoriasis was named “white mange” (*bai bi*) in “*Wai Ke Da Cheng*”.

According to the textbook entitled “Surgery of Traditional Chinese Medicine” [11], psoriasis is attributed to long stagnation of pathogenic *wind* and *heat* resulting from exposure to exogenous wind, cold, or heat, or from internal factors of emotional injury and stagnation of *qi*, and obstruction of the meridians and collaterals that leads to *qi stagnation* and *blood stasis*. If the meridians and collaterals are obstructed by exogenous wind, dampness, and heat toxin, the condition can also affect the joints.

Unlike Western medicine, TCM does not emphasize disease classification, but instead classifies a patient by their *Zheng* (TCM syndrome or pattern). Consequently, two patients with the same disease may be classified into different *Zhengs* according to their symptoms and signs, and different treatment protocols will be given according to the identified *Zhengs*. However, two patients suffering from different diseases may have the same pathologic *Zheng* and require the same treatment protocol.

Studies on psoriasis in TCM have reported approximately five different *Zheng* classifications. Zhang *et al.*[12] and Wang *et al.*[13] classified *blood heat* and *blood dryness* as the two main *Zheng* classes. Zhang *et al.*[11] found *blood stasis* as the third *Zheng*, while Wang *et al.*[13] found *dampness heat* and *blood stasis* as the third and fourth *Zhengs*. Wu *et al.*[14] identified *yin deficiency* as one of the *Zhengs* in psoriasis. Zhang *et al.*[12], Wang *et al.*[12], and Wu *et al.*[14] classified *blood dryness* as one *Zheng* class of psoriasis. However, Wang *et al.* and Wu *et al.* disagreed on the other classes. Li *et al.*[15] used the five viscera to classify psoriasis *Zhengs*, while Li [16] based his classification on *wind dryness* with three different *Zheng* combinations. All of these studies were descriptive and did not report statistical analyses of clusters or classes.

Amidst the variation in classifications, a national diagnosis standard for the classification of psoriasis was announced in 1994 [17], and it has not been updated since. The standard classification includes three *Zhengs*: (1) *wind heat* and *blood dryness*; (2) *wind* and *dryness* due to *blood deficiency*; and (3) *blood stasis*. Most of the previous studies have been based on expert opinions, without any study protocols. There were also inadequate attempts to classify the patients.

A systematic checklist [18] was developed for reproducible data collection and statistical analysis. The patients could be further clustered and assigned to groups of *Zhengs*.

Latent class analysis (LCA) is a statistical method for identifying unmeasured class memberships among subjects using categorical or continuous observed variables, or both [18]. LCA attempts to achieve data reduction by classifying the subjects into one of K unobserved classes based on observed data, where K is fixed and known. Such latent classes are identified by their relationships with the observed variables [19] for disease subtypes or diagnostic subcategories.

Thus, LCA would be applicable to clustering of psoriasis in TCM. This study aims to perform an LCA to cluster psoriatic patients into various *Zheng* groups based on the application of a checklist of TCM symptoms and signs.

## Methods

### Study design and setting

A cross-sectional study was carried out at Yunnan Provincial Hospital of Traditional Chinese Medicine and the First Affiliated Hospital of Kunming Medicine University. This study was performed during a 12-month period from October 2010 to September 2011, after receiving approval from the Ethical Committee Board of the Faculty of Medicine, Prince of Songkla University.

### Study subjects

The psoriatic subjects were aged over 10 years, and recruited from inpatients and outpatients of the Dermatology and Rheumatism Departments of the two hospitals. The sample size was initially set at 500 [20] to fit a checklist of 96 items (the development of the checklist is described below). The exclusion criteria were patients having communication difficulties, severe psychiatric disorders, or cognitive problems.

Patients with severe dysfunction of organs and those receiving retinoid, methotrexate, or glucocorticosteroid treatments within 1 month were also excluded, because their symptoms and signs would be altered by the dysfunctions and disease treatments.

### The TCM standard checklist of symptoms and signs of psoriasis

The process of the checklist development was described in detail in a previous article [21]. Briefly, a three-round Delphi study was conducted by 16 experts (seven from Beijing and nine from Yunnan) between July and September 2010. A draft checklist was created by literature review of articles and textbooks. In the end, there were 96 items (eight domains) of psoriatic symptoms and signs in TCM in the checklist. The eight domains included (1) color, (2) shape, (3) type of skin lesion, (4) associated factors, (5) physical expression, (6) tongue substance and tongue coating, (7) pulse, and (8) living environment. The psoriasis symptom items were rated “yes” or “no”. Notes on symptoms and signs and pulse palpation were completed by the first author (XY).

### Patient recruitment

The selected psoriatic patients were informed of the objectives and search procedures of the study and signed consent forms themselves or through their guardians if they were adolescents. The TCM checklist of symptoms and signs was completed. All of the patients had a confirmed diagnosis by exhibiting typical skin lesions and other physical signs. A skin pathological examination confirmed the cases in doubt. The color and texture of the skin lesion and tongue were observed under ambient light in the examination room. Personal health behaviors, such as smoking, drinking, and associated diseases, were assessed during an interview. Hypertension was verified using a mercury sphygmomanometer and a history of anti-hypertensive use was taken. Diabetes mellitus was evaluated by history alone.

### Statistical methods

Descriptive statistics were used to summarize the characteristics of the patients regarding their symptoms and signs. LCA was used to classify the subjects based on the variables of symptoms and signs in TCM collected in the checklist. The patients were allocated to classes based on the maximum estimate of posterior probability of cluster membership. Model solutions were evaluated using the Bayesian Information Criterion (BIC), which has been shown to be a robust indicator of the preferred K class solution [22]. Lower BIC values indicated a better model fit. The Vuong-Lo-Mendell-Rubin likelihood ratio (VLMR-LR) test goodness-of-fit index, which considers parsimony, was used to compare the null hypothesis of k-1 clusters with alternative clusters [23]. The significance tests used to compare the characteristics among the classes were the chi-square test for categorical variables and the Kruskal–Wallis test for continuous variables.

R 2.12.2 software (R Foundation, Austria) [24] was used to perform the descriptive statistics. Mplus software 5.0 (Muthén & Muthén, USA) [25] was used for the LCA. A class was defined as the group into which patients were identified by the prominent probability in their symptom and sign manifestations present in the checklist [18].

The composition of each class (and class name) was independently evaluated by five independent dermatology experts. Their opinions were then cross-checked. Any inconsistent opinions were settled by discussion among these experts to determine the final *Zheng* classes.

## Results

Five hundred and seven patients were recruited. Their characteristics are summarized in Table 1. The percentage of males was slightly higher than that of females. Most of the patients were Han in ethnicity, and 83.4% were outpatients. The patient ages ranged from 11.5 to 74.8 years. High school as the highest education level accounted for 50.3%. The patients had varied duration and severity of disease, and the majority were psoriasis vulgaris cases. The median duration of disease was 5.6 years, and the interquartile range was 2.0 to 11.9 years. More than half of the patients had no reported associated diseases. However, hypertension (8.1%), diabetes (3.4%), and gastrointestinal diseases (8.9%) were not uncommon.

**Table 1:** Demographic, social, and clinical characteristics of the 507 patients

**Variable**	**Number (n = 507)**	**Percentage**
Sex		
Male	294	58.0
Ethnicity		
Han	462	91.1
Others	45	8.9
Age (mean ± standard deviation)	37.0 ± 15.8	—
Type of psoriasis		
Vulgaris	461	90.9
Erythrodermic	17	3.4
Pustular	16	3.0
Arthritic	13	2.6
Marital status		
Single	179	35.3
Ever-married	328	64.7
Associated diseases		
Hypertension	41	8.1
Coronary heart disease	8	1.6
Diabeties	17	3.4
Hepatitis	9	1.8
Gastrointestinal diseases	45	8.9
Other diseases	113	22.3
No disease	274	53.9
Psoriasis duration (years), Median (IQR)	5.6 (2.0, 11.9)	—
Progression phase	441	87.0
Stable phase	49	9.7
Remission phase	17	3.4
Smoking	162	32.0
Drinking	133	26.2
Season of exacerbation		
Spring	109	21.5
Summer	97	19.1
Autumn	115	22.7
Winter	186	36.7

### Number of classes based on the LCA

Items on types and shapes of skin lesions were excluded from the LCA because they represented the expressions of the psoriatic diseases caused by groups of *Zhengs*, and were not considered to be symptoms and signs of the underlying *Zheng*. Items occurring in less than 2% of the patients were also excluded because they were negligible to clustering.

After the LCA, 50 items of symptoms and signs were retained in the models. Table 2 shows the values for the BIC and VLMR-LR tests for models with various numbers of classes and their distributions. The model with three classes gave the lowest BIC, and was better than the model with two classes (VLMR p-value = 0.037). The model with four classes was not significant (p = 0.70). Therefore, the three-class model was chosen as the best fitted model. Using this model, the percentages of subjects falling into classes 1, 2, and 3 were 35.1%, 34.7%, and 30.2%, respectively.

**Table 2:** Results of the latent class analysis: model fit indices comparing different class solutions

**Index**	**Number of class (k)**			
	**2**	3*	**4**	**5**
BIC	31936	31788	31987	31904
VLMR LR test (p-value)	0.0004	0.037	0.7025	0.9799
Proportion of each class	47.3%	35.1%	24.1%	21.9%
	52.7%	34.7%	21.1%	16.0%
		30.2%	21.9%	31.4%
			32.9%	9.1%
				21.6%

*Best fitted model.

Within each class, items with probability higher than 0.6 were sorted according to the four examination methods of TCM (inspection, auscultation and olfaction, interrogation, and palpation). Six symptoms and signs were common to all three classes (white coating, predilection heavy and greasy, intermittent itching, string pulse, dry taste and thirst, and bluish sublingual varicose veins).

All five independent TCM dermatology experts agreed to these three *Zhengs*. Based on the high frequencies of various symptoms and signs (marked by asterisks in Table 3) in each *Zheng*, the experts concluded that class 1 was dominated by *dampness-heat Zheng*, class 2 by *blood heat Zheng*, and class 3 by a combined *yin deficiency* and *blood dryness Zheng*.

**Table 3:** Prominent items (probability higher than 0.6) in each class

**Diagnosis method**	**Class 1 (n = 178)**	**Class 2 (n = 176)**	**Class 3 (n = 153)**
	***Dampness-heat Zheng***	***Blood heat Zheng***	***Yin deficiency *** **and *blood dryness Zheng***
Inspection	Darker skin color* (73.1%)	Garnet skin color* (62.3%)	Pink skin color* (92.2%)
		Darker skin color (60.2%)	
		Sore throat* (87.6%)	
	Sore throat (76.4%)	Pharyngeal wall	—
		Follicular* (67.8%)	
		Fissured tongue (63.3%)	
	Red tongue* (60.7%)	Crimson tongue* (77.0%)	Red tongue* (61.5%)
	Flabby tongue (62.9%)		Red tip of tongue (65.1%)
	Teeth-printed tongue (65.7%)		Thin tongue* (66.7%)
	Yellow tongue coating* (72.7%)		Thin tongue coating* (81.5%)
	Thick tongue coating*(80.6%)		Less tongue coating* (92.3%)
	Greasy coating* (98.5%)		
	—	Scanty dark urine* (67.9%)	—
Auscultation and olfaction	Fetid breath (62.5%)	—	—
Interrogation	Predilection pungent (72.0%)	—	Predilection pungent (77.2%)
	Attacking on depression and morose (71.8%)	—	Predilection cold and raw food (62.3%)
	—	—	Anxiety and irritability* (73.5%)
	—		Frequent dreams (65.8%)
		—	Constipation* (65.7%)
	—	—	Aggravated after coldness (72.3%)
	—	—	Slight itching (61.5%)
	—	—	Overexertion* (61.8%)
Palpation	Slippery pulse* (68.9%)	—	Thready pulse* (64.5%)
	—	—	Rapid pulse* (66.8%)

*Essential item predominating in each *Zheng* class.

The latent clusters obtained in this study were groups of subsets of latent variables that usually occurred together. For example, class 1 in Table 3 consists of a group of variables that can be interpreted as *dampness* (flabby tongue, teeth-printed tongue, yellow, thick, and greasy tongue coating, and slippery pulse) and another group representing *heat* (darker skin color, sore throat, red tongue, fetid breath, and predilection pungent). Patients in this class manifest both *Zhengs* together and, in such cases, *dampness-heat Zheng* is used to name this class. A thorough discussion of LCA in TCM and interpretation of latent clusters was performed by Zhang *et al.*[26]. Seven symptoms and signs (garnet, darker skin color, sore throat, pharyngeal wall follicular, fissured tongue, crimson tongue, and scanty dark urine) were present in the second class with mean invasion of the blood phase by pathologic *heat. Blood heat Zheng* defines this cluster. Even though this cluster reflects *heat* like the first cluster, the symptoms and signs of *heat* were more prominent and not concomitant with *dampness Zheng*. In fact, *dampness* could lessen the expression of *heat Zheng* in the first class, but the patients still suffered from both *dampness* and *heat Zhengs*. Eight symptoms and signs representing *yin deficiency* (red tongue, thin tongue, red tip of tongue, thin tongue coating, predilection cold and raw food, anxiety and irritability, frequent dreams, and rapid pulse) were involved in the third cluster. In the third cluster, four symptoms and signs (pink skin color, less tongue coating, overexertion, and aggravated after coldness) could be interpreted as *blood dryness Zheng*. There are four symptoms and signs (constipation, slight itching, thready pulse, and less tongue coating) in the third cluster, which are known to occur in combined *yin deficiency* and *blood dryness Zheng*.

### Characteristics of psoriatic patients among the three classes

The characteristics of the psoriatic patients among the three classes are summarized in Table 4. Winter was the worst season for all three *Zheng* classes Exacerbations of lesions in winter were most pronounced in *dampness-heat Zheng*. However, patients with *yin deficiency* and *blood dryness Zheng* were likely to have lesions all year round with a better disease course in summer, while those with *dampness-heat* and *blood heat Zhengs* tended to have lesions that would subside when the temperature increased. The *blood heat Zheng* class had the highest proportion of males and the highest median age. The patients classified as having *yin deficiency* and *blood dryness Zheng* were less likely to be hospitalized. Smoking and alcohol drinking were more prevalent in the *blood heat Zheng* group. The associations between psoriasis class and other diseases, such as hypertension and gastrointestinal disease, were not strong with P-values of 0.06 and 0.07, respectively.

**Table 4:** Characteristics of the psoriatic patients among the three classes

**Variable**	**Class 1 (n = 178)**	**Class 2 (n = 176)**	**Class 3 (n = 153)**	***P *** value*
	***Dampness-heat Zheng***	***Blood heat Zheng***	***Yin deficiency *** **and *blood dryness Zheng***	
Gender				
Male	97 (54.5)	129 (73.3)	70 (45.8)	< 0.01
Female	81 (45.5)	47 (26.7)	83 (54.2)	
Seasons				
Spring	28 (15.7)	41 (23.3)	40 (26.1)	0.045**
Summer	36 (20.2)	32 (18.2)	29 (18.9)	
Autumn	39 (22.0)	35 (19.9)	41 (26.8)	
Winter	75 (41.1)	68 (38.6)	43 (28.1)	
Age, Median (IQR)	34.5 (24.2, 47.3)	39.9 (28, 53.6)	32.2 (22.4, 40.7)	< 0.001
Ethnicity				
Han	165 (92.7)	159 (90.3)	137 (89.5)	0.667
Others	13 (7.3)	17 (9.7)	16 (10.5)	
Type of patient				
In-patient	47 (26.4)	33 (18.8)	5 (3.3)	< 0.001
Out-patient	131 (77.6)	143 (81.2)	148 (96.7)	
Smoking				
Yes	49 (27.6)	79 (44.9)	34 (22.2)	< 0.001
No	129 (72.4)	97 (55.1)	119 (77.8)	
Drinking				
Yes	33 (18.5)	65 (36.9)	35 (22.9)	0.003
No	145 (81.5)	111 (63.1)	118 (77.1)	
Associated diseases				
Hypertension	15 (8.4)	19 (10.8)	7 (4.6)	0.065
Gastrointestinal disease	8 (4.5)	17 (9.7)	20 (13.1)	0.074
Diabetes mellitus	7 (3.9)	6 (3.4)	4 (2.6)	0.610

*Chi square test.

**Kruskal Wallis test.

*IQR*, inter-quartile range.

## Discussion

This study classified psoriatic patients into three groups, based on the three main *Zhengs* they suffered, *i.e.*, “*dampness-heat*”, “*blood heat*”, and “*yin deficiency* and *blood dryness*”. Nevertheless, there were six miscellaneous symptoms and signs common to all three *Zheng* classes without specificity for any one class, namely (1) predilection for a heavy or greasy diet, (2) white tongue coating, (3) bluish sublingual varicose veins, (4) intermittent itching, (5) dry taste and thirst, and (6) *string pulse*.

Zhang *et al*. [26] suggested latent tree models and diagnosis in TCM. This study is the first to use a standard checklist of symptoms and signs and a proper statistical analysis to investigate the *Zhengs* in psoriasis disease. There is known to be an assumption of mutual independency for the manifested variables for LCA. However, this assumption is often violated to some extent, especially when there are a large number of variables [27].

In contrast to Zhang *et al.*[12] and Wang *et al.*[13], in which statistical techniques for clustering of symptoms and signs were not used, our results suggested that *blood dryness* should be classified together with *yin deficiency*. Wu *et al.*[14] also identified *yin deficiency* as one of the *Zhengs* in psoriasis, but this was combined with other *Zhengs*, and not with *blood dryness* as found in our study. Zhang *et al.*[12], Wang *et al.*[13], and Wu *et al.*[14] classified *blood dryness* as one *Zheng* class of psoriasis, similar to our study.

The LCA employed a model-based clustering approach, placing patients experiencing similar patterns of symptoms and signs into the same class [28]. Different findings in *Zheng* may be caused by (1) researchers using different checklists, (2) clinicians having different training and experience, and (3) variation in epidemiological patterns of *Zheng* arising through variation in the population characteristics, geography, and time periods. Based on the theory of TCM, in which personal constitutions are the foundation of disease development, there might be some variation in the *Zheng* diagnosis among populations living in different geographic regions and climates [29,30].

Based on TCM theory, *Zheng* was associated with the seasons. Cui *et al.*[31] reported seasonal diversity of TCM *Zhengs* in 609 cases of acute diarrhea. Tan *et al.*[32] reported four *Zhengs* in 698 hypertension cases. The occurrence of *liver fire flaming-up Zheng* was common in spring (102 cases) and relatively rare in summer (16 cases). The other three *Zhengs* included *yin deficiency* and *yang excess* (92 cases), *phlegm-dampness* accumulation (82 cases), and *yin-yang deficiency* (64 cases). Our study showed that the *Zhengs* in psoriasis were associated with seasons. *Dampness-heat Zheng* was predominant in summer (48.9%) and autumn (30.8%), and *cold dampness Zheng* was dominant in winter (53.6%) and spring (35.6%). Guo *et al.*[33] reported the onset and exacerbation of psoriasis with *liver gallbladder dampness-heat Zheng* associated with spring.

Our results showed different *Zhengs* from the previous studies, which might arise through differences in geography. Geographic influence on *Zheng* has been known in studies on irritable bowel syndrome, showing clear differences between patients in the south of the five ridges area and those in the northeast area of China [34]. For viral hepatitis, TCM doctors in the southern and northern parts of China used different theories [35]. *Dampness-heat Zheng* is always associated with *spleen*, *stomach*, *liver*, and *gallbladder*[36]. In previous studies, *dampness-heat Zheng* was commonly found in pustular psoriasis and psoriatic arthritis [37,38]. However, our patients were mainly psoriasis vulgaris cases, and this association could not be tested. *Dampness-heat Zheng* was common in our study in Yunnan, where the diet generally includes spicy, heavy, greasy, and pungent foods, and such a diet might cause *dampness-heat*[39]. However, *dampness-heat* was reported to be common in inner-Mongolia (16% were *dampness-heat* of *spleen* and *stomach*) by Li *et al.*[15] and Beijing by Wang *et al.*[13].

*Blood heat* was the second most common *Zheng* in this study. The mechanism of *blood heat* has been described as invasion of *heat* into the *blood* path resulting in disease [11]. Usually, rapid and string pulse is the manifestation of *blood heat*. However, the character of the pulse was not classified in class 2 (*blood heat Zheng*) because the LCA detected string pulse in all three classes and rapid pulse was more predominant in class 3 (*yin deficiency* and *blood dryness*) than in class 2. Although both pulse characteristics were common to *blood heat Zheng*, they were not useful for the *Zheng* class differentiation. *Blood heat Zheng* was mentioned in the national criteria for *Zheng* diagnosis of psoriasis and also reported in 53.8% of patients by Zhang *et al.*[12]; in 36.7% by Wang *et al.*[13], and reported as *heart blood heat* by Li *et al.*[15] (25.0%) and by Li *et al.*[16] as combined*wind dryness* and *blood heat. Blood heat Zheng* was the most commonly reported *Zheng* in psoriasis studies in China.

The combined *yin deficiency* and *blood dryness Zheng* was the third group in our list. These two factors might interact and influence the psoriasis course in the long term. This class was a combination of symptoms and signs of the two subgroups of *Zhengs*. Red and thin tongue, red tip of tongue, anxiety and irritability, frequent dreams, predilection cold and raw, and thready and rapid pulse are symptoms and signs of *yin deficiency*[40]. *Liver kidney yin deficiency* was reported by Wu *et al.*[14] (21 of 200 cases). Another subgroup of symptoms and signs of *Zheng* consisting of pink color, thin and less tongue coating, and thready pulse are the main manifestations of *blood dryness*[41]. *Blood dryness Zheng* has been more commonly reported (27.4%, Zhang *et al.*[12]; 6.0%, Wu *et al.*[14]; 36.7%, Wang *et al.*[13]) than *yin deficiency Zheng*.

There were six symptoms and signs that could not be assigned to any one of the three *Zhengs*. These were common to all three classes, and their significance and underlying pathologic mechanisms require further investigation.

Unlike previous reports on the classification of psoriasis, which were based on single or only a few experts’ opinions, ours was based on a consensus checklist, systematic data collection for a large sample size, reliable statistics, and final agreement on the *Zheng* clustering among 16 experts from different hospitals.

To our knowledge, there has been no established standard for *Zheng* classification in TCM for psoriasis. Even though our clustering reached expert consensus, the level of accuracy of this clustering could not be assessed because variation in the *Zheng* classification might occur in different geographic and climate environments and populations. Thus, our results need to be verified in different settings in China. Furthermore, this study was carried out among moderate to severe psoriasis patients who attended specialized hospitals. Studies among mildly affected patients might elucidate a wider spectrum of psoriasis *Zheng* clustering.

## Conclusions

In this study, 507 psoriasis patients could be clustered into three *Zhengs*, which had different associated factors. The first *Zheng*, *dampness-heat*, was associated with exacerbation in winter, the second, *blood heat*, was associated with male sex, old age, smoking, and alcohol drinking, and the third, *Yin deficiency* and *blood dryness*, was associated with a mild clinical course without necessity for hospital admission.

## Competing interests

The authors declare that they have no competing interests.

## Authors’ contributions

XY, VC, and HS designed the study. XY, VC, and SL performed the statistical analyses. XY, VC, and HS wrote the manuscript. EY, XO, and JY facilitated the data collection in China. All authors read and approved the final manuscript.
